# Highlights from the 10th Breast, Gynaecological and Immunotherapy International Cancer Conference (BGICC), 18–19 January 2018, Cairo, Egypt

**DOI:** 10.3332/ecancer.2018.820

**Published:** 2018-03-22

**Authors:** Hesham El-Ghazaly, Adel Aref, Nermeen Bahie-eldin

**Affiliations:** 1 Breast and Gynaecological International Cancer Society (BGICS), Cairo 11757, Egypt; 2 Clinical Oncology department, Ain Shams University, Cairo 11566, Egypt; 3 University of Adelaide Medical School, Adelaide 5000, Australia

**Keywords:** breast cancer, gynaecological cancer, immunotherapy, BGICC, BGICS

## Abstract

During the 10th Breast, Gynaecological and Immunotherapy International Cancer Conference (BGICC), which was held on 18 and 19 of January, 2018, in Cairo, Egypt, around 100 international, regional and national experts presented the latest updates in breast cancer, gynaecological cancers and immunotherapy in oncology. Through this report, we will try to highlight the important data and consensus issues that were discussed during the conference.

## Introduction

The Breast, Gynaecological and Immunotherapy International Cancer Conference (BGICC), which was held during the period of 18 to 19 January, 2018, in Cairo, Egypt, was celebrating its 10th round this year. The BGICC is one of the international multi-disciplinary conferences dedicated to fostering the science of Breast and Gynaecological oncology and improving the care of cancer patients and their families throughout the world. It is the biggest cancer conference in Africa and the Middle East. In this report, we will present the highlights of the important data presented during the 10th BGICC, with special consideration to triple negative breast cancer (TNBC), management of Her2 positive disease, breast cancer prevention, the BGICC consensus sessions and the BGICC education courses.

## Advances in management of triple negative breast cancer

The management of TNBC is one of the hottest topics in breast cancer. Professor Joyce O’Shaughnessy (Baylor Charles A. Sammons Cancer Center, USA) discussed new advances in the management of TNBC. The role of platinum-based agents in TNBC is still evolving; however, it is so far proving to be effective. Professor O’Shaughnessy discussed the data from the recent studies showing that platinum-based chemotherapy leads to durable overall response rate (ORR) of 11.7% in patients with TNBC even in the BRCA wild subtype.

The role of PD-L1 inhibitors was also discussed, with special consideration to the results from the KEYNOTE-086 study [1]. Data from KEYNOTE-086 showed that PD-L1 inhibitor (Pembrolizumab) as monotherapy leads to 5% ORR in metastatic TNBC irrespective of the PD-L1 expression. A recent large phase-I study showed that another PD-L1 inhibitor (Atezolizumab) is safe in the first line setting with durable response rate of 10% [2].

Professor O’Shaughnessy also talked about new potential targets in the management of TNBC. The effectiveness of oral AKT inhibitor (Ipatasertib) was explored in the LOTUS study as a potential target in TNBC. The addition of Ipatasertib to Paclitaxel in the first line setting leads to an increase in the progression free survival [3]. Targeting Her2 is a new approach in management TNBC. Around 17% of TNBC are Her2 enriched, in which there is activated Her2 mutation without gene amplification [4]. Neratinib is an oral irreversible inhibitor of Her1, Her2 and Her4 receptors [5]. Professor Joyce O’Shaughnessy discussed the recent data on Neratinib, which showed an increase in pathological complete response (pCR) in Her1 and Her2 enriched TNBC.

Professor Banu Arun (University of Texas, MD Anderson Cancer Center, USA) talked about the new advances in neoadjuvant and adjuvant treatments in TNBC. Professor Arun discussed the impact of adding platinum-based chemotherapy on pCR in TNBC. Updated results of the CALBG 40603 study [6] showed that the addition of carboplatin improved the pCR significantly but this was not translated into survival benefit. However, the GeparSixto study [7] showed that addition of platinum-based chemotherapy increases the pCR significantly and this time, it was translated into improvement in overall survival. Professor Arun emphasised that, although the toxicity profile of platinum-based treatment should be considered, the addition of platinum-based treatment in the setting of TNBC is of great importance when rapid and significant response rate is required.

## Advances in Her2 positive breast cancer

Professor Matti Aapro [International Society of Geriatric Oncology (SIOG) and, Multinational Association of Supportive Care in Cancer (MASCC), Switzerland] discussed the heterogeneity in Her2 positive breast cancer with concentration on the role of microenvironment and tumour infiltration lymphocyte in response to anti-Her2 treatment.

Professor Aapro highlighted two important points, which are management of brain metastasis progression with anti-Her2 treatment and overcoming ‘Financial’ resistance to the anti-Her2 targeted agents. Data from the 4^th^ Advanced Breast Cancer Meeting, Lisbon, which was held in Lisbon, Portugal, in 2017, recommended that anti-Her2 treatment should be changed only if brain metastasis is the only site of progression post anti-Her2 treatment, and no local treatment is available. This represents a very important recommendation in the management of brain metastasis progression in Her2 positive patients.

Professor Aapro also discussed the new term ‘Financial resistance’. This year, the BGICC was considering breast cancer management in low- and middle-income countries. Despite the progress in breast cancer research and the improvement of clinical response and patient’s survival with the new targeted agents, the cost of these agents still represent a major barrier in their clinical implementation. Professor Aapro discussed the role of bio-similars and its potential benefit in low- and middle-income countries.

The benefit of long- versus short-term adjuvant anti Her2 treatment was also discussed. Preliminary results from ‘The synergism or long duration’ study [ClinicalTrials.gov identifier NCT00593697] showed that after 5 years follow-up, 9 weeks of adjuvant trastuzumab is not ‘NON inferior’ to 1 year, with Disease free survival (DFS) of 88 % versus 90.5% for the 9 weeks and 12 months, respectively (hazard ratio, 1.39; 90% confidence interval, 1.12–1.72). There was no significant difference in overall survival or distant disease-free survival. This is a very important result and may increase the clinical administration of Trastuzumab in areas of low and middle income.

Another important session was a debate around the addition of Pertuzumab for all versus selected patients in the adjuvant setting for Her2 positive patients. Professor Javier Cortes (Vall d'Hebron Institute of Oncology, Spain) discussed the benefits of adding Pertuzumab to Trastuzumab in the adjuvant setting with highlighting the improvement in disease free survival (DFS) and overall survival (OS) that was achieved. Professor Cortes was in favour of the combination for all patients. However, Professor Hope Rugo (UCSF Medical Center, USA) advised that patient selection should be considered before using the anti-Her2 combination, taking into consideration the marvellous improvement in DFS and OS with Trastuzumab only, which exceeded 98%. Professor Rugo was in favour of keeping the combination for selected patients after considering the toxicity, response required and cost.

## The first BGICC-SITC immunotherapy consensus

Professor Hesham El-Ghazaly, head of the BGICS and the secretary general of the BGICC, presented the results of the 1st BGICC-SITC immunotherapy consensus. This consensus was released during the 9th BGICC. The consensus came with important concepts in immunotherapy including the importance of sticking to the clinical design of each study and that the ‘Approval of one of checkpoint inhibitors does not permit the use of the others in the same indication’ and that ‘Immunotherapy optimally should not be used outside of the approved indications except within clinical trials’. Again, the consensus panel highlighted the importance of cost effectiveness studies with an emphasis on the statement that ‘Cost effectiveness studies should be encouraged in the context of immunotherapy’.

The panel advised that ‘There are different cut off levels of expression of PD-L1 as indication of immunotherapy depending on type and site of tumour as well as the line of treatment (first versus second)’. The panel felt that the available data still points towards the usage of Computed tomography scan or Magnetic resonance imaging rather than Positron emission tomography scan for assessment of response to immunotherapy.

The consensus recommended that ‘Immune related response criteria’ are the preferred criteria in response assessment to immunotherapy’.

## CDK 4/6 inhibitors in breast cancer

Professor Hope Rugo discussed the recent advances in the role of cyclin D-dependent kinase 4/6 inhibitors in breast cancer. Recent results of the PALOMA-2 study, which explore the efficacy of combing palbociclib plus letrozole versus placebo plus letrozole [8], showed that the palbociclib extended the progression free survival from 14 to 28 months. Professor Rugo discussed the subset analysis, which showed that palbociclib was efficient across all the subgroups of the study cohort; however, there was a trend of more benefit in luminal A and B subgroups. The quality of life was maintained in the palbociclib arm. In addition, she highlighted the toxicity profile of the combination, which should be considered in patient selection.

## BGICC Consensus for management of advanced ovarian cancer

The BGICC international expert panel discussed the best management of advanced ovarian cancer. The panel came to a consensus about some of the hot topics in the management of advanced and recurrent ovarian carcinoma. Some of the important recommendations of the panel were the ‘importance of upfront cyto-reductive surgery with the aim of leaving residual less than 5 mm’. In addition, the panel recommended to start with neo-adjuvant chemotherapy in cases which are not fit for surgery or when no expert experience is available. The panel did not advise on the use of heated intra-peritoneal chemotherapy and felt that more data is required before setting a standard of care.

## Breast cancer prevention

Professor Cheng-har Yip (Subang Jaya Medical Centre/Parkcity Medical Centre, Malaysia and University of Malaya, Malaysia) discussed breast cancer prevention in low- and middle-income countries. As breast cancer prevention was the pillar of the 10th BGICC, Professor Yip gave an excellent introduction on breast cancer prevention in low- and middle-income countries. Professor Yip showed the difference in breast cancer epidemiology between developed and developing countries, especially the younger age of presentation in developing countries. Professor Yip discussed the risk factors of breast cancer, especially the modifiable risk factors (diet, exercise, alcohol intake, exposure to Estorgen) and the fact that most of the studies did not show a well-established role in breast cancer prevention, leaving bilateral mastectomy as the only reliable method for breast cancer prevention. In addition, most of these factors are already not very dominant in low- and middle-income countries. Moving forward from this point came the importance of early detection and breast screening in decreasing the incidence of breast cancer in low- and middle-income countries, which could have an impact on reducing breast cancer specific mortality.

Professor Diana Bowser (Brandeis University, USA) discussed the role of developing evidence-based health policies with special consideration to developing countries. Professor Bowser discussed the models used in different countries, with some examples from African and Asian countries. Professor Bowser highlighted the importance of such models in improving the outcome of disease management especially in areas of limited resources.

## BGICC consensus for breast cancer prevention and screening in low- and middle-income countries

A group of 40 international experts in breast cancer have discussed the best modalities of breast cancer screening and prevention in low- and middle-income countries. This consensus was done in collaboration with WHO-EMRO, National cancer higher committee, Harvard University, UICC, NCCN-MENA and BGICS.

Over the important recommendations that came out of this session were ‘the ultimate need for regional breast cancer prevention guidelines’, the importance of increasing the role of general practitioners and family physicians in prevention and awareness campaigns. The panel advised that ‘breast cancer awareness campaigns should be promoted to all adult age groups’, not only those who are at risk; however, the panel advised ‘that early detection programs should be designed and planned with priority to reach the target population’. An important pillar of this consensus is that the population education and clinical examination by clinical staff can improve breast cancer incidence and mortality in areas of limited resources with a lack of radiological facilities. The panel thought that there is a need for a specific risk assessment tool that considers breast cancer and socio-economic criteria in areas of limited resources.

## BGICC interactive education courses

During the BGICC, a number of educational, interactive courses took place. Each course was by separate registration, with only 100 attendees per each course. The aim of these courses was to help young oncologists, surgeons and gynaecologists in gaining experience through an interactive session which is moderated and presented by the top experts in each field.

During the radiotherapy course, Professor F. Geara (American University of Beirut, Lebanon) highlighted the management of acute reaction in patients receiving hypo-fractionation radiotherapy treatment. Professor R. Orecchia (European Institute of Oncology, Italy) discussed tailoring radiotherapy according to molecular profiling and recent techniques in radiotherapy. Professor Dzugashvili (Madrid Oncology Institute, Spain) discussed the most recent guidelines of radiotherapy in endometrial cancer and real-life clinical application of these guidelines.

During the pathology course, Professor Stolnicu (University of Medicine Târgu Mureș, Romania) talked about the new classification proposal, a new human papilloma virus (HPV)-led screening and classification protocol for women with endocervical adenocarcinoma. She described the accuracy and uptake of Human Papilloma virus (HPV) screening for gynaecologic disease, and also its utility in testing for other virus-induced cancers.

During the surgery course, Professor Wickman (Karolinska University Hospital, Sweden) discussed eligibility criteria for onco-plastic surgery and the increased public awareness of risk markers including BRCA as a driver of surgical interventions. She discussed the latest in surgical and implant technology, and the importance of patient quality of life as a clinical outcome.

In the Gynaecology course, Professor Kehoe (Institute of Cancer and Genomic Sciences, University of Birmingham, UK) talked about the DESKTOP III trial comparing the efficacy of additional tumour de-bulking surgery versus chemotherapy alone for recurrent platinum-sensitive ovarian cancer. Recent results are showing improvement in survival and quality of life with the investigation arm. Professor Grénman (Turku University Hospital, Finland) discussed preoperative identification of low-/high-risk endometrial cancer, noting the variation in treatment prospects between and within facilities. In addition, she described molecular classification of endometrial cancers as a promising means of guiding treatment options for clinicians and patients.

## Conclusion

Very important data was discussed during the 10th BGICC, Cairo, Egypt. During this conference, new advances in the management of breast and gynaecological cancers were presented. In addition, there was excellent coverage on areas of debate, especially in immunotherapy. Three BGICC special consensuses were released this year, for immunotherapy, management of advanced ovarian cancer and for breast cancer prevention in low- and middle-income countries.
